# Dispersal Patterns of Coastal Fish: Implications for Designing Networks of Marine Protected Areas

**DOI:** 10.1371/journal.pone.0031681

**Published:** 2012-02-15

**Authors:** Antonio Di Franco, Bronwyn M. Gillanders, Giuseppe De Benedetto, Antonio Pennetta, Giulio A. De Leo, Paolo Guidetti

**Affiliations:** 1 Laboratory of Conservation and Management of Marine and Coastal Resources, Dipartimento Di Scienze Etecnologie Biologiche E Ambientali (DiSTeBA), University of Salento, Lecce, Italy; 2 Southern Seas Ecology Laboratories, School of Earth and Environmental Sciences, University of Adelaide, South Australia, Australia; 3 Laboratorio di Analisi Chimiche per l'Ambiente e i Beni Culturali, Dipartimento dei Beni delle Arti e della Storia, University of Salento, Lecce, Italy; 4 Department of Environmental Science, University of Parma, Parma, Italy; Swansea University, United Kingdom

## Abstract

Information about dispersal scales of fish at various life history stages is critical for successful design of networks of marine protected areas, but is lacking for most species and regions. Otolith chemistry provides an opportunity to investigate dispersal patterns at a number of life history stages. Our aim was to assess patterns of larval and post-settlement (i.e. between settlement and recruitment) dispersal at two different spatial scales in a Mediterranean coastal fish (i.e. white sea bream, *Diplodus sargus sargus*) using otolith chemistry. At a large spatial scale (∼200 km) we investigated natal origin of fish and at a smaller scale (∼30 km) we assessed “site fidelity” (i.e. post-settlement dispersal until recruitment). Larvae dispersed from three spawning areas, and a single spawning area supplied post-settlers (proxy of larval supply) to sites spread from 100 to 200 km of coastline. Post-settlement dispersal occurred within the scale examined of ∼30 km, although about a third of post-settlers were recruits in the same sites where they settled. Connectivity was recorded both from a MPA to unprotected areas and vice versa. The approach adopted in the present study provides some of the first quantitative evidence of dispersal at both larval and post-settlement stages of a key species in Mediterranean rocky reefs. Similar data taken from a number of species are needed to effectively design both single marine protected areas and networks of marine protected areas.

## Introduction

Dispersal is the process by which living organisms expand actively or passively the space or range where they live and is one of the fundamental life-history traits affecting the dynamics of spatially structured populations [1]. Individual dispersal basically involves departure from the initial site, movement between sites (transience) and arrival in a new site [2]. Collecting information about patterns of dispersal at population level is critical not only in terms of basic ecological knowledge, but also for applied issues [3], [4]. For example, designing effective marine protected areas (hereafter MPAs) or networks of MPAs requires information on scales of dispersal at different life stages [3]–[7]. Such information is crucial to assess connectivity among MPAs [8], or between MPAs and surrounding unprotected areas [9]. Accurate measurements of dispersal distances may thus assist effective management and conservation policies [10].

In spite of their crucial relevance, data on larval and juvenile dispersal of coastal fishes are scarce, including in the regions where MPAs are particularly numerous (e.g. Mediterranean Sea, >100 MPAs; [11]). The scarcity of data is attributable to the difficulty in obtaining basic information (e.g. artificial tagging is of limited use due to the small size of early stages and high rates of mortality, [12]–[14] but see [15]). The available evidence (gained through tagging studies, otolith chemistry and population genetics) suggests that larval dispersal may be shorter than previously suspected (i.e. up to 200 km) and that juvenile dispersal is highly variable and limited to few tens of km (see [12] for a review). Estimating dispersal distances of early stages thus remains one of the greatest challenges in marine ecology [7]. Otolith chemistry provides a potential opportunity to investigate dispersal patterns at a number of life history stages. Profiles of the dispersal history of an individual can be derived from chemical information stored in the otoliths [16]–[17] (but see [18]). Otoliths incorporate into their calcium carbonate matrix both minor and trace elements as they grow [19] with some elements (e.g. Sr and Ba) incorporated at rates related to ambient concentrations [20]–[21] or in relation to other environmental variables (e.g. temperature and salinity, see [19] for further details). Otoliths, therefore, may represent a natural biological tag [19], [22] that can be used to investigate dispersal history of fishes [22]–[24]. Assaying otoliths of post-settlers collected along a stretch of coast and identifying groups based on elemental signatures in otolith cores (the portion of the otolith originating at birth and thus related to the natal origin of fish) can provide information about the spatial scale of larval dispersal [25]–[26]. In short, provided that different groups supposedly corresponding to different natal origins [26]–[27] can be identified through otolith analysis (see [28] for a large scale analysis on *D. sargus sargus*), the scale of larval dispersal can be inferred. This could be achieved on the basis of *a*) the distance among different sampling sites that were replenished by a single source [26] and *b*) the number of potential source populations within the sampled area.

Moreover, evaluating “site fidelity” of juvenile fish between settlement and recruitment to adult populations and/or the distance travelled between settlement and recruitment sites, can provide information about juvenile spatial dispersal after settlement. This goal can be achieved, first, by characterizing the elemental signatures of the portion of the otolith formed just after settlement (the portion that is chemically characterized by the site where the fish settled) from post-settlers sampled at multiple sites. Then, a similar analysis (i.e. on the same portion of the otolith) can be done on recruits collected in the same sites. Post-settlement spatial dispersal between settlement and recruitment to adult populations can thus be inferred by looking at the chemical match between post-settlers and recruits at various sites (analogous to the approach used to track adult specimens to the estuaries where they recruited previously, see [29]). Similar chemical composition in otoliths of post-settlers and recruits collected from the same site implies that juvenile fish recruited in the same site where they settled. This approach can have crucial implications in assessing the role of MPAs in retaining or exporting juvenile fish: knowledge about patterns of dispersal can thus help to shed light on the potential ability of MPAs in improving fisheries in unprotected areas through both larval and juvenile export [4], [30].

This study focuses on the dispersal patterns of early life stages of a coastal fish, the white sea bream *Diplodus sargus sargus* (Linnaeus 1758), distributed in the Eastern Atlantic, Mediterranean and Black Seas. This species was selected as a model species due to its ecological [31], [32] and economic importance for many professional and recreational fisheries [33]. This fish usually inhabits the littoral zone in shallow waters down to about 50 m [34] and shows an increase in density and size due to protection from fishing (i.e. inside MPAs, see [35]–[36] for evidence from the Mediterranean Sea). Adults are relatively sedentary [37] and demersal, and produce eggs and larvae that develop in the pelagic waters for a period ranging from 16 to 28 days [28], [38], [39]. Post-larvae then metamorphose and settle in shallow (less than 2 m depth) coastal benthic habitats (mainly small bays with mixed sand and rock [34]) at about 1.0 cm TL [40]. Juveniles recruit when approximately 6–7 cm in size, ∼5 months after settlement [40], [41]. Information about early life history of this fish including dispersal is scarce, even though such information is crucial to better understand population dynamics and for management issues. The aim of the present study, therefore, is to investigate and assess spatial scales of dispersal of larvae and post-settlers of *D. sargus sargus* along the south-western Adriatic coast.

## Methods

### Sampling collection and study area

Larval and post-settlement dispersal were assessed at two different spatial scales: at a larger scale (∼200 km) we investigated natal origin of fish and at a smaller scale (∼30 km) we assessed “site fidelity” from post-settlement to recruitment to the adult population. These scales (referred to hereafter as ‘sites’ and ‘regions’) represent key scales of dispersal for larval and juvenile coastal fishes (see [12] for a review about dispersal). The regional scale focused at a scale in which the available evidence suggested that post-settlement dispersal occurred at [12] and with where recruits were found along the coast (see below).

**Figure 1:**
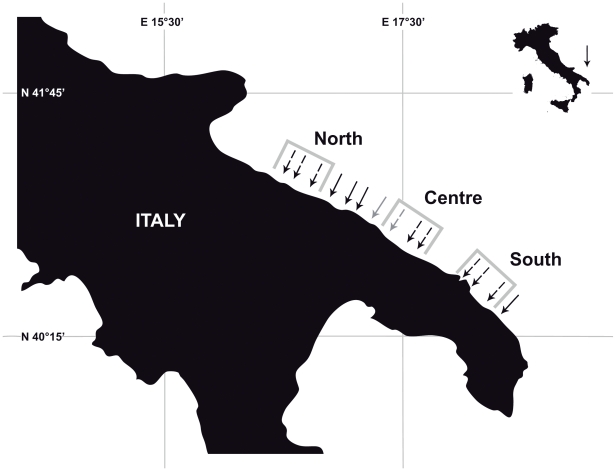
Study area. Arrows represent the 14 sampling sites (in the text numbered progressively from the northern one to the southern one) for settlers and the dotted arrow represents which of these sites are also sampling sites for recruits. Grey arrows indicate sites located inside TGMPA (number 7 and 8). Polygons indicate the three regions considered for the post-settlement dispersal analysis.

Post-settlers of *D. sargus sargus* (i.e. 1–1.5 cm TL) were collected just after the settlement peak, which was assessed through visual surveys conducted from 8–15^th^ June 2009. Fish were collected from 14 sites along ∼200 km of the Apulian Adriatic coast (Fig. 1). Two of these sites were inside the Torre Guaceto Marine Protected Area (TGMPA) and twelve sites outside (six northward and six southward, along the Adriatic Apulian coast up to 100 km from the border of TGMPA). At each site, 10 specimens were collected with a hand-net. Previous findings about multivariate components of variation in otolith chemical composition of the same species found that the variability associated with the factor “otolith” (i.e. variability among fishes within a single site) was much less than the among-site variability [28] therefore this sample size was deemed suitable.

Approximately 4–5 months after settlement (October-November), when peak recruitment was detected through visual census surveys (authors' unpublished data, but see also [40]) recruits of white sea bream (i.e. 6–8 cm TL) were collected. Therefore, post-settlers and recruits collected in the present study belonged to the same cohorts. Collection of recruits was carried out in 9 of the 14 sites where the post-settlers were previously collected. The remaining 5 sites had few, if any, recruits of *D. sargus sargus*. The 9 sites where recruits were collected were grouped into three to represent three regions (i.e. stretches of coastline ∼30 km long), named North, Centre and South regions (Fig. 1). In the ‘Centre’ region, one of the three sites was located inside the TGMPA, while the remaining two were located 8–12 km (one north and one south) from the TGMPA borders. At each site, 6–10 recruits were collected, providing a total of 85 specimens.

After collection both post-settlers and recruits were immersed in an ice slurry (<5°C) to minimize suffering and then stored in 95% ethanol [27]. The experimental fishing activity was performed in strict accordance with the authorization protocol provided by Italian Minister of Agriculture, Foods and Forestry Politics (Permit Number: 0011267-2010). Methods were consistent among sites, as well as for post-settlers and recruits, to avoid any bias in subsequent analyses.

### Sample preparation and analysis

In the laboratory, one sagitta was removed from each specimen, cleaned of soft tissue using plastic dissecting pins and then mounted sulcus side up onto a glass slide using crystal bond, previously tested to ensure it was not a source of contamination. Otoliths were polished with 3 µm and 1 µm Imperial lapping film to expose inner growth layers for analysis. We chose not to polish the otolith to the core and to leave material above it in order to ensure the core was not removed during pre-ablation procedures, which potentially allowed us to sample all the material associated with the core [42]. After polishing with lapping film, otoliths were rinsed and sonicated for 10 minutes in ultra-pure water.

**Figure 2:**
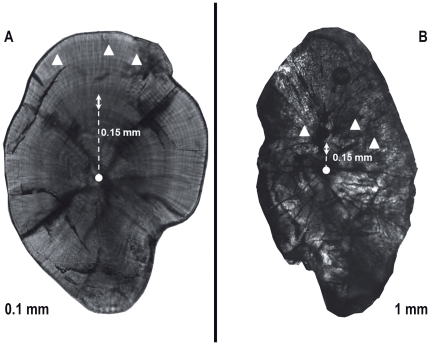
Otoliths of a) settler and b) recruit specimen. Photos are taken at different magnification: 200× for a) and 40× for b). Dots indicate cores, triangles indicate ablations in juvenile region, double arrowed lines indicate settlement marks. Dotted lines indicated distances between core and settlement mark.

Otoliths of post-settlers were analyzed for the chemical composition of both the core (in order to acquire information about natal origin) and the post-settlement portion (i.e. the ten increments after the settlement mark). Otoliths of recruits were only analyzed for the chemical composition of the post-settlement portion (Fig. 2). In post-settlers we isolated the material associated with the core using three discrete vertical pits of 30 µm (identified previously as the approximate size of the cores in *D. sargus sargus*, [28]) from the surface of the otolith through the visible core. The spike in Mn:Ca was used as an indicator of the core location, as previous studies have reported elevated Mn concentrations in the core [43], [44] and therefore just one out of the three pits (the one showing at least 3-fold higher Mn:Ca concentration than surrounding material, [42]) was considered in subsequent analysis. A Mn:Ca spike could not be detected in around 15% (21 samples) of the core samples of post-settlers; these samples were excluded from further analysis of natal origins. In the post-settlement portion of both post-settlers and recruits we ablated three horizontal pits and all three were considered in subsequent analysis in order to account for within-otolith variability (see [28] for further details). The otoliths were placed in the ablation chamber and viewed remotely on a computer screen where the area for ablation was selected. The laser was focused on the sample surface and fired through the microscope objective lens using a spot size of 30 µm. Each run generally consisted of 40 s acquisition: 10 s blank to correct for background which was subtracted from each sample, 10 s ablation (laser at 65% power, about 6 J/cm^2^) resulting in a pit about 10 µm deep and 20 s for washout. Prior to analysis, samples were pre-ablated to remove any surface contamination (laser at 50% power). Helium gas was flushed into the ablation cell to reduce the deposition of ablated aerosols and to improve signal intensities. The ablated aerosol was then mixed with argon before entering the ICP torch.

All otoliths were analyzed using a Thermo Elemental ×7 inductively coupled plasma mass spectrometer (ICP-MS) coupled to a NewWave Research UP213 with aperture imaging laser ablation (LA) system. External calibration was performed with two Standard References Materials (SRM) from National Institute of Standards and Technology, NIST 610 and NIST 612. Calcium was used as an internal standard to account for variation in ablation and aerosol efficiency. All the 9 elements analyzed (^24^Mg, ^55^Mn, ^66^Zn, ^88^Sr, ^138^Ba, ^208^Pb, ^7^Li, ^57^Fe, ^59^Co) were expressed as ratios relative to ^44^Ca.

Detection limits were calculated from the concentration of analyte yielding a signal equivalent to 3× the standard deviation of the blank signal for each of the elements (see Table 1). Mean estimates of precision (%RSD, relative standard deviation) and accuracy based on 154 replicate measurements of NIST 610 and NIST 612 were calculated (Table 1). The recorded values of Li, Fe and Co were consistently below the detection limits and therefore excluded from the analyses. Due to values below the detection limit, Mn was excluded from analyses of the post-settlement portion of the otolith.

**Table 1:** Estimates of precision, accuracy and limits of detection (LOD).

Element	NIST 610%RSD	NIST 612% RSD	% Accuracy NIST 610	% Accuracy NIST 612	LOD
Mg:Ca	8.10	14.5	101	107.2	0.05597
Mn:Ca	5.36	9.73	100.99	112.31	0.035373
Zn:Ca	7.37	10.34	99.94	120.7	0.024758
Sr:Ca	4.70	9.43	100.57	92.51	0.0320972
Ba:Ca	8.90	9.84	101.43	88.39	0.005346
Pb:Ca	13.29	19.07	99.26	122.89	0.004217

LOD are given in mmol mol^–1^. Values for %RSD (% relative standard deviation) and % accuracy.

### Statistical analyses

To determine the number of potential source populations, the core elemental concentrations of post-settlers (as a proxy for identifying the existence of single or multiple areas of origin [42]) were analyzed by cluster analysis. The similarity profile permutation test (SIMPROF) procedure was used to determine which clusters were significantly different at the 5% level. Elemental/Ca ratios that contribute to the significant differences among groups (i.e. natal origin) were identified using similarity percentage (SIMPER).

Because homogeneity in otolith chemical composition may simply reflect environmental similarity, we evaluated potential spatial variability in otolith chemical composition. For this purpose we used permutational multivariate analysis of variance (PERMANOVA) to test for differences between the 14 sampling sites by analysing the otolith edge of post-settlers (i.e. post-settlement portion laid down just before capture). ‘Site’ (Si) was treated as a random factor (fourteen levels), ‘Otolith’ (Ot) as a random factor nested in (Si) (ten levels). There were three replicate ablations for each otolith (total n = 420).

Three canonical analysis of principal coordinates (CAP, [45]) and jackknife cross validation (% of correct classification), one per region, were performed on the elemental data from juvenile portion of the post-settler otoliths to assess how accurately they were classified to sites where they were collected in each region. Recruits were assigned to settlement sites (i.e. the sites where the post-settlers were collected) through linear discriminant functions previously parameterized with post-settlers otoliths. Centroids per specimen for both post-settlers and the juvenile portion of otoliths of recruits (i.e. centroid of the three replicates for each specimen) were calculated and used for CAP analysis. Recruits were assumed to come from an un-sampled settlement site when their distance from the centroid of the group (i.e. post-settlers belonging to a settlement site) to which they were assigned was higher than the largest distance between post-settlers inside the group (i.e. from the same settlement site); in this case recruits were not assigned to any settlement site. Statistical analyses were run using Primer 6 PERMANOVA+software package.

## Results

**Figure 3:**
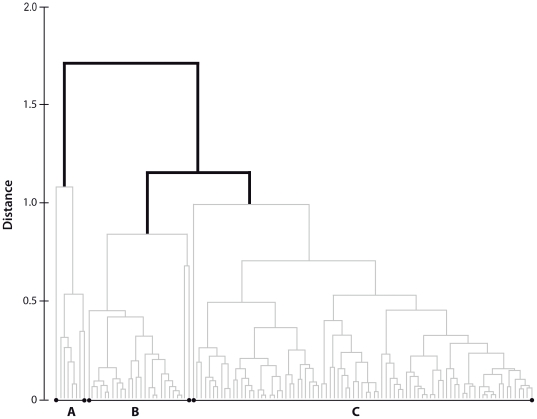
Classification of settlers' otolith cores. Thin grey lines indicate non-significantly different samples, thick dark lines separate significantly different groups (named by letters A, B and C).

**Figure 4:**
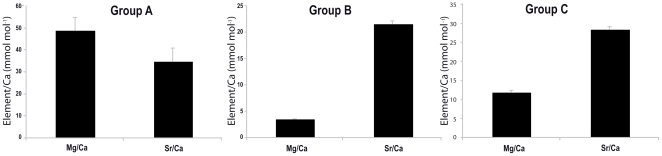
Average element/calcium ratio (± standard error) in the core region for the groups identified by SIMPROF analysis.

Three statistically different groups (Fig. 3) were found for the core samples of post-settlers, suggesting three different natal origins (i.e. spawning areas). Most post-settlers were part of group named C (71.2%), with a smaller percentage in group B (22%) and group A (6.8%). The elemental/Ca ratios contributing most to differences among groups were Mg:Ca and Sr:Ca (about 100% of the total dissimilarity in pairwise comparison among groups, SIMPER analysis); Zn:Ca, Pb:Ca, Ba:Ca and Mn:Ca had little influence in determining differences between groups. The three groups differed in the elemental ratios of Mg:Ca and Sr:Ca in the otoliths (PERMANOVA p<0.01 for both elemental ratios). Group A was characterized by relatively high concentrations of both Mg:Ca and Sr:Ca (Fig. 4). Group B was characterized by medium Sr:Ca and low Mg:Ca concentrations, while Group C was distinguished by high Sr:Ca and intermediate Mg:Ca concentrations (Fig. 4). Group A comprised specimens mostly fished in sites located south of TGMPA, whereas groups B and C were a mixture of specimens from all the sampling sites (i.e. only two spawning areas replenished all sampling sites). Post-settlers collected in the two sites inside TGMPA belong either to groups B and C suggesting that the two settlement sites sampled at TGMPA were replenished by two different spawning areas (Table 2).

**Table 2:** Percentage of post-settlers from one of the three groups based on core signatures, in each sampling site.

Sampling site	% of fishes from group A	% of fishes from group B	% of fishes from group C
**Site 1**	0	0	100
**Site 2**	0	33.3	66.7
**Site 3**	0	100	0
**Site 4**	0	25	75
**Site 5**	2.5	10	87.5
**Site 6**	0	0	100
**Site 7**	0	66.7	33.3
**Site 8**	0	71.4	28.6
**Site 9**	12.5	0	87.5
**Site 10**	33.3	66.7	0
**Site 11**	0	22.2	77.8
**Site 12**	9.1	54.5	36.4
**Site 13**	30	20	50
**Site 14**	0	22.2	77.8

Sites were numbered progressively from the northern one to the southern one.

The chemical composition of the juvenile portion of post-settlers was significantly different among the sampling sites (PERMANOVA, pseudo-f: 4.4872, p<0.01). Significant differences among otoliths were also found (pseudo-f: 6.1871, p<0.01) suggesting within-site differences among individuals.

**Figure 5:**
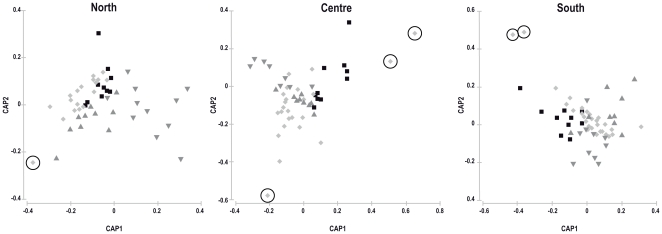
CAP analyses of trace elements (Mg, Zn, Sr, Ba, Pb) from otoliths of settlers. Each panel refers to one of the three regions (North, Centre and South) sampled in the present study. In every panel each of the three sampling sites considered is represented by a different symbol. New samples (representing otoliths of recruits collected in the region considered) are depicted by grey symbols. Dotted circles indicate samples not assigned to any group (see material and methods for further details).

For post-settlement dispersal, 76.6% (Pillai's trace = 1.04, p<0.001) of post-settlers from the northern region were correctly classified to collection site in cross-validation of CAP analysis. When the linear discriminant functions built with post-settlement fingerprints of post-settlers were applied to recruits, one recruit (3% of the total recruits) was not assigned to any of the settlement sites, therefore was considered to have settled outside the sampling region (Fig. 5). In the northern region, a total of 97% of recruits showed otolith chemical composition that matched those of post-settlers at 2 out of the 3 sites (respectively 37% and 60% to each of the two sites) in the region, while no recruits were assigned to the third settlement site. Considering just the recruits assigned to settlement sites, approximately 49% of recruits were found in the same site where they settled, ∼18% recruited to sites 6–8 km away and a further 18% recruited to sites 20 km away. The remaining ∼14% moved ∼30 km between settlement and recruitment (Table 3).

**Table 3:** Percentage of post-settlement dispersal in each of the three regions considered.

	North	Centre	South
**0 km**	48.8	22.7	28.5
**∼8 km**	18.5	NA	32.1
**∼15 km**	NA	63.6	NA
**∼20 km**	18.5	NA	36.1
**∼30 km**	14.2	13.6	3.3

NA = not available in the region.

For the central region 69.9% of fish were correctly classified to their respective sites (Pillai's trace = 0.958, p<0.001); three recruits (about 12% of the total recruits) were not assigned to any of the settlement sites (Fig. 5). In the central region 46% of the recruits showed chemical composition of the otolith that matched that of post-settlers collected at the site located inside TGMPA, while 42% of recruits showed chemical composition of otoliths that matched those of post-settlers from the other two sites within the region. In this region, considering just the recruits assigned to settlement sites, 22.7% of post-settlers did not move from settlement sites, 63.6% and 13.6% moved ∼15 and 30 km, respectively, from their settlement sites (Table 3). 28% of recruits from TGMPA showed chemical composition of otoliths that matched those of post-settlers from TGMPA. The remaining 72% of recruits from TGMPA were assigned to one of the other two sites, whereas 22% and 16% of post-settlers from the unprotected sites did not move from settlement sites.

In the southern region 70.5% (Pillai's trace = 0.956, p<0.001) of samples were correctly classified to the area that they were collected from. Two recruits (about 6% of the total) were not assigned to any of the settlement sites (Fig. 5). About 75% of recruits were assigned to a single settlement site, while 15.6% and 3.4% of recruits were assigned to the other 2 settlement sites. Around 28.5% of recruits recruited to the same settlement site where they were collected, whereas ∼32%, 36% and 3.3% moved ∼6–8, 20 and 30 km away, respectively, from their settlement site (Table 3).

Overall (considering recruits assigned to settlement sites) in terms of post-settlement dispersal, 33.7% of recruits settled in the same site in which they were then collected, whereas the remainder appeared to move away from settlement sites: 18.2% moved 6–8 km, 18.2% moved 15 km and 19.4% moved about 20 km away; 10.3% moved 30 km away from their settlement site.

## Discussion

For many coastal fishes dispersal occurs at an early life-history stage (e.g. larval phases, [46]). Larval dispersal is the primary mode of migration that connects spatially discrete fish sub-populations, while dispersal at other life stages is often considered of little importance in determining connectivity. Connectivity in marine metapopulations is thus typically equated with dispersal patterns of larvae [14], [46]. This hypothesis, however, is largely untested and information about dispersal at other life stages (e.g. juvenile) is lacking (but see [47]). The scales at which connectivity takes place at the various life stages for each fish are largely unknown. As populations of many species decline [48], accurate measurements of dispersal distances are needed to assist with effective management and conservation policies [10], [49].

In this study we provide evidence that, for the coastal fish *D. sargus sargus*, larval dispersal occurs at the scale of 100–200 km, although 200 km was the maximum spatial scale analysed. This conclusion is inferred by taking into account the maximum distance between sampling sites replenished by each spawning area (i.e. natal origins identified in terms of different groups or clusters). In our study, single spawning areas were found to supply settlers (used as a proxy for larval supply) to sites spread over ∼200 km of coastline. It is possible that a single group of core signatures (i.e. natal origin) may represent multiple natal origins since homogeneity may simply reflect environmental similarity among sites. This potential bias can be reasonably excluded in the present study considering the high spatial variability recorded among sampling sites. This spatial variability in chemical fingerprint is likely related to spatial differences in seawater chemistry, temperature and salinity. Unfortunately no data about these variables are currently available for the study area, although the coastal area is characterized by a multitude of runoffs (i.e. streams and other little freshwater inputs as water discharges) and spans approximately 1 degree of latitude.

Post-settlement dispersal took place over the maximum distance we considered in the present study (ca. 30 km), with about a third of post-settlers recruiting in the same sites where they settled. Post-settlement dispersal and connectivity were directed both from MPA to unprotected areas and vice-versa. Dispersal at post-settlement stage could be affected by reef morphology with longer dispersal associated with continuous reefs (i.e. rocky reefs without any relevant discontinuity such as large sandy bays). In the present study, reefs were continuous and dispersal could be reduced in patchy or isolated conditions (i.e. small isolated rocky structures). From this perspective it would be crucial to evaluate dispersal at different degrees of reef patchiness to identify how reef patchiness influences the spatial scale of fish dispersal at different life stages.

All these estimates are considered conservative due to the scales of sampling adopted however this study, provides some of the first quantitative evidence of dispersal at both larval and post-settlement stages of a littoral fish.

Marine currents and long pelagic larval stages for most organisms creates a high potential for long-distance dispersal, despite relatively sedentary adult phases [10], [49]. However, recent evidence shows short-distance larval dispersal [15], [50]–[53] and sharp genetic breaks [54], [55] in species thought to have potentially high dispersal. Dispersal at sea, therefore, may actually be surprisingly lower than expected: 10 to 100 km for invertebrates and 50 to 200 km for fish (see [12] for a review).

Little information is available on dispersal of juvenile (post-larval) stages [12]. Different ranges of dispersal have been described: extremely reduced (i.e. 100 m, [56]), few kilometres [57], [58] to 10 kilometres or more [59], [60]. These papers encompass a wide range of fish (from different environments), different sizes (from 2 to 15 cm) and a wide range of durations of life phase (from 1 to 5 months). [61] assessed dispersal in a fish from the same family as *Diplodus*, of a similar size and across a similar duration (i.e. 5 months), finding juvenile fish dispersing up to about 30 km. The only evidence about juvenile dispersal of *D. sargus* suggests very low dispersal [62]. This evidence, however, arose just from observation of a single individual over a very short time (i.e. 2 days). More information is available about dispersal between juvenile and subadult stages, arising primarily from otolith chemistry (see [63] for findings about multiple coastal fishes).

Our study highlights individual variability in dispersal at both larval and juvenile stages. For the larvae this evidence is likely related to differences in oceanographic patterns: larval dispersal depends not only on the biology of the species (e.g. hatching date, pelagic larval duration and larval swimming behaviour), but also on the regional to local oceanographic features (e.g. directional currents, topographic gyres and cross shelf currents) and hydrological parameters (temperature, salinity) [64]. The dynamics of ocean currents at scales appropriate to larval dispersal are very complex, highly variable even on a very short time scale and not fully characterized yet [6].

The analysis of otolith chemistry suggested that the majority of post-settlers sampled were probably generated in one single spawning area (C, see results for details), a substantially smaller but still significant fraction in a second spawning area (B) and a very small fraction of post-settlers, mostly fished in sites located south of TGMPA, were generated in a third spawning area (A). The nature of our data does not allow us to identify where these three spawning areas are located along the coastline. Nevertheless another study (Di Franco *et al.* submitted) has shown that, within this study area, sites in TG-MPA host the highest density of spawners. It is therefore possible that these sites inside TGMPA represent one of the contributing spawning areas (A,B or C) or even the highest contributing spawning area (C). Moreover, as the third spawning area contributed mostly to fish settled in sites located south of TGMPA and as the dominant sea currents along this coast are mainly directed from north to south [65], it can be hypothesized that this third spawning area (A) is located in the south region of the study area. This is in agreement with the lack or scarcity of settlers from this spawning area in the north and centre region of the study area. Whether the second spawning area could be also located within TGMPA or at north of the protected area it is hard to say on the basis of available data. More investigation, possibly making use of artificial tagging or genetic analyses, is required to disentangle this issue.

In the present study post-settlement dispersal at the individual level was highly variable, with some fish not dispersing at all and other fish dispersing over 30 km. This difference in terms of dispersal could be related to fish personality as defined by [1], e.g. boldness, sociability or aggressiveness. Some studies have shown a positive correlation among boldness (a measure of the level of exploration in unfamiliar habitat) and dispersal distance [66]–[67]. Moreover, the interaction among individuals seems to influence potential dispersal: a classic idea is that less aggressive, subordinate individuals are forced to disperse by aggression from more dominant individuals (see [1] for a review) suggesting that individuals may disperse when in higher densities. Therefore dispersal might have a significant density dependent component. As a consequence, differences in dispersal among protected and unprotected areas may be found, given that fish densities are often higher inside MPAs [35], [68]. Our study suggests that retention (i.e. the fraction of recruits remaining in areas where they supposedly settled) is substantially higher in sites within the northern region (on average about 50%), where density of settlers is much lower (Di Franco et al submitted), than in sites within the central and southern regions (on average about 22% and 28% respectively), where the densities are indeed higher (i.e. density is comparable among central and southern regions and about 6 times higher than in northern region, Di Franco *et al.* submitted). This hypothesis however requires further testing.

Very few data are available that are derived from direct observations of early stage dispersal distances [12]. Direct measurements of dispersal are needed to better understand connectivity in a network of MPAs. MPAs are intended to serve community and ecosystem functions, and these functions involve species with many different dispersal patterns, most of which are unknown. Determining the optimal spacing of MPAs within a network requires knowledge about how far larvae, juveniles and adults regularly disperse or move, which could allow decisions about how close MPAs need to be to be effectively connected [7].

The approach adopted in the present study is a useful start [7] since it provides information about dispersal of a key species in Mediterranean rocky reefs. Such information, if available for a number of fish species, could contribute to optimizing the design of single MPAs (e.g. in terms of size) and/or the distance apart that MPAs are spaced to ensure effective networks.
